# Characterization of antigen-presenting properties of tumour cells using virus-specific cytotoxic T lymphocytes

**DOI:** 10.1054/bjoc.1999.1081

**Published:** 2000-03-21

**Authors:** D C J Spierings, E Agsteribbe, J Wilschut, A Huckriede

**Affiliations:** Laboratory of Molecular Virology, Department of Medical Microbiology, University of Groningen, Ant. Deusinglaan 1, Groningen, AV, 9713, The Netherlands

**Keywords:** tumour, antigen presentation, immunotherapy, influenza virus

## Abstract

Immunotherapy of tumours by induction of tumour-specific cytotoxic T-lymphocytes (CTLs) will only be effective for tumours with a functional antigen processing and presentation machinery. However, many tumours are known to down-regulate expression of major histocompatibility complex (MHC) class I molecules and/or to impair antigen processing. It is therefore desirable to evaluate the ability of a given tumour to present antigenic epitopes before developing an immunotherapy protocol. In this study we have used influenza virus as a tool to determine the antigen-presenting capacities of the murine neuroblastoma C1300 cell line NB41A3, a frequently used model for human neuroblastoma. Immunofluorescence analyses revealed low and moderate expression of MHC class I molecules D^d^and K^k^respectively. Nevertheless, infected NB41A3 cells were lysed efficiently by influenza-specific CTLs. These results demonstrate that all steps of the antigen-processing pathway function properly in the NB tumour cells, and that the limited MHC class I expression suffices for efficient recognition by CTLs. In addition, lysis of the NB tumour cells shows that the cells are susceptible to CTL-induced apoptosis, a pathway that is often impaired in tumour cells. These characteristics make neuroblastoma a suitable target for immunotherapy. The presented assay allows evaluation of various immunological properties of tumour cells and, thus, represents a valuable tool to assess whether a given tumour will be susceptible to immunotherapy or not. Copyright 2000 Cancer Research Campaign. © 2000 Cancer Research Campaign


*British Journal of Cancer* (2000) **82**(8), 1474–1479
© 2000 Cancer Research Campaign
DOI: 10.1054/ bjoc.1999.1081, available online at http://www.idealibrary.com on IDEAL®

### Characterization of antigen-presenting properties of tumour cells using virus-specific cytotoxic T lymphocytes

**DCJ Spierings, E Agsteribbe*, J Wilschut and A Huckriede**

Laboratory of Molecular Virology, Department of Medical Microbiology, University of Groningen, Ant. Deusinglaan 1, 9713 AV Groningen, The Netherlands

**Summary** Immunotherapy of tumours by induction of tumour-specific cytotoxic T-lymphocytes (CTLs) will only be effective for tumours with a functional antigen processing and presentation machinery. However, many tumours are known to down-regulate expression of major histocompatibility complex (MHC) class I molecules and/or to impair antigen processing. It is therefore desirable to evaluate the ability of a given tumour to present antigenic epitopes before developing an immunotherapy protocol. In this study we have used influenza virus as a tool to determine the antigen-presenting capacities of the murine neuroblastoma C1300 cell line NB41A3, a frequently used model for human neuroblastoma. Immunofluorescence analyses revealed low and moderate expression of MHC class I molecules $D^d$ and $K^k$ respectively. Nevertheless, infected NB41A3 cells were lysed efficiently by influenza-specific CTLs. These results demonstrate that all steps of the antigen-processing pathway function properly in the NB tumour cells, and that the limited MHC class I expression suffices for efficient recognition by CTLs. In addition, lysis of the NB tumour cells shows that the cells are susceptible to CTL-induced apoptosis, a pathway that is often impaired in tumour cells. These characteristics make neuroblastoma a suitable target for immunotherapy. The presented assay allows evaluation of various immunological properties of tumour cells and, thus, represents a valuable tool to assess whether a given tumour will be susceptible to immunotherapy or not. © 2000 Cancer Research Campaign

**Keywords:** tumour; antigen presentation; immunotherapy; influenza virus

Within the context of the theory of immunological tumour surveillance, the immune system is considered to play an important role in the control and elimination of malignant cells. It is now well established that tumour rejection can be mediated by lymphocytes, most notably, $CD8^+$ cytotoxic T lymphocytes (CTLs). Consequently, many immunotherapy approaches have been developed to enhance the immune response to tumours by directly activating tumour-specific CTLs. Since immune recognition by CTLs requires the presentation of processed peptides in association with major histocompatibility complex (MHC) class I molecules, the success of these cancer vaccination strategies is dependent on the ability of the malignant cells to endogenously process and present target epitopes on their cell surface. However, malignant transformation is frequently associated with escape of tumour cells from immune recognition. Defects in the endogenous processing function may result in a down-regulated presentation of specific antigens in the context of surface MHC class I molecules on tumour cells (Marincola et al, 1994*a*; Ferrone and Marincola, 1995; Hicklin et al, 1999) and, thus, in resistance to CTL-mediated immune recognition (Darrow et al, 1989; Restifo et al, 1993; Rivoltini et al, 1995; Seliger et al, 1997). Three different types of antigen processing and/or presentation defects in tumour cells have been documented (reviewed by Khanna, 1998). Firstly, loss of surface MHC class I expression, caused by structural alterations or dysregulation of the MHC class I molecules, probably represents one of the most important strategies used by tumour cells to evade CTL-mediated recognition. A second immune escape mechanism involves abnormalities in processing of endogenous antigens. For example, a down-regulated expression of LMP2 and LMP7, two subunits of the multicatalytic proteasome complex, blocks the generation of peptide epitopes. Finally, complete deletion of coding sequences or point mutations in peptide transporter genes resulting in loss of TAP-1 and TAP-2 expression inhibits the transport of peptide epitopes from the cytoplasmic compartment into the endoplasmic reticulum. Tumour cells can exhibit either one or a combination of these defects to avoid recognition by CTLs. Since all these immune escape mechanisms will impair the effectiveness of CTL-based immunotherapy approaches, it is highly desirable to establish the antigen-presenting capacities of a given tumour before setting out to develop such a therapy.

Immunohistology and fluorescence-activated cell sorting (FACS) analysis, two assays based on antibody binding, have been widely used to analyse the presence of MHC class I molecules on tumour cells (Garrido et al, 1993; Marincola et al, 1994*b*). However, drawbacks of these assays are the restricted sensitivity and the fact that only expression of MHC class I molecules is measured rather than the capacity of the tumour cells to process and present endogenous antigens. Specific lysis by high-affinity CTLs can be induced by only a small number (< 10–100) of ligands per target (Demotz et al, 1990; Harding and Unanue, 1990;

*Received 9 September 1999*
*Revised 8 November 1999*
*Accepted 11 November 1999*

*Correspondence to:* A. Huckriede

*This paper is dedicated to the memory of Dr. Etienne Agsteribbe, who deceased 11 February 1999

Christinck et al, 1991; Sykulev et al, 1996). Consequently, a poor or negligible expression of MHC class I molecules as determined by immunohistology or FACS analysis, does not necessarily indicate complete absence of recognition by CTLs.

The present study describes a method to directly determine the antigen-presenting properties of (tumour) cells using influenza virus as a tool. In this approach, tumour cells which, after virus infection, process and present viral antigens properly, will be recognized and lysed by influenza-specific CTLs while absence of lysis indicates the presence of defects in the antigen-processing machinery. To evaluate the potential of this influenza virus-based method we used the murine neuroblastoma (NB) C1300, a well-established model for human disease (Ziegler et al, 1997). NBs are poorly immunogenic and characterized by low expression of MHC class I antigens (Lampson et al, 1983). Nevertheless, we observed that influenza-infected NB41A3 cells are efficiently lysed by influenza-specific CTLs. This result indicates that early steps in antigen processing are not impaired in NB41A3 cells, that a small number of antigen-MHC class I complexes is sufficient to allow recognition by specific CTLs, and that NB tumour cells are vulnerable to CTL-mediated cytotoxicity.

### MATERIALS AND METHODS

### Cell lines

The murine C1300 neuroblastoma cell line, clone NB41A3, originating from an A/J mouse (H-2$D^dK^k$), the L929 fibroblasts (H-2$K^k$), and the mastocytoma cell line P815 (H-2$D^d$) were obtained from the American Type Culture Collection (ATCC, Rockville, MD, USA). Cells were cultured in RPMI-1640 medium (Gibco-BRL, Breda, The Netherlands) supplemented with 10% heat-inactivated fetal calf serum (FCS; PAA Laboratories GmbH, Austria) and 1 mM glutamine.

### Antibodies

Monoclonal antibody (mAb) reactive with mouse MHC class I H-2$K^k$ (mouse IgG2a) was purchased from ATCC (HB-16 16-1-11N hybridoma cell line). mAb to MHC class I H-2$D^d$ (mouse IgG2a, MCA 1055) was obtained from Serotec (Apeldoorn, The Netherlands). Fluorescein isothiocyanate (FITC)-conjugated goat anti-(mouse IgG) was purchased from Nordic Immunological Laboratories (Tilburg, The Netherlands).

### Treatment of cells with interferon α

NB41A3 cells were cultured in fresh medium containing 750 U $ml^{-1}$ murine recombinant interferon γ (IFN-γ; Gibco-BRL). The cells were incubated for 3 days and washed in medium before use.

### Immunofluorescence

IFN-γ-stimulated or non-stimulated NB41A3 cells ($5 \times 10^5$) were washed twice in cold phosphate-buffered saline (PBS) containing 1% bovine serum albumin (BSA; Sigma, Zwijndrecht, The Netherlands) and incubated with monoclonal anti-H-2$D^d$ or anti-H-2$K^k$ antibodies in pre-tested concentrations for 30 min at 0°C. P815 and L929 cells were used as positive H-2$D^d$ and H-2$K^k$ controls respectively. Cells were then washed three times and were further incubated for 30 min at 0°C with FITC-conjugated goat anti-(mouse IgG) antibodies. After washing, the cells were resuspended in the same medium plus propidium iodide to exclude dead cells during analysis. Stained cells were analysed on an Epics-Elite flow cytometer (Coulter-Electronics, Hialeah, FL, USA). The percentage of positive cells was determined by comparison of the fluorescence labelling to control cells labelled with secondary antibody alone.

**Table 1** Flow cytometric analysis of the expression of MHC class I molecules $D^d$ and $K^k$

| Cell line | Mean fluorescence | |
|---|---|---|
| | MHC I $D^d$ | MHC I $K^k$ |
| P815 | 183.7 | ND |
| L929 | ND | 63.9 |
| NB41A3 | 24.1 | 38.5 |
| IFN-γ-stimulated NB41A3 | 86.5 | 99.7 |
| Infected NB41A3 (2.5 h) | 21.3 | 36.8 |
| Infected NB41A3 (6 h) | 25.1 | 45.4 |

ND, not determined.

### Induction of CTLs

Six- to 8-week-old female A/J mice (H-2$D^dK^k$) were obtained from Harlan (Zeist, The Netherlands) and maintained in our animal facilities. For sensitization in vivo, mice were given intraperitoneal (i.p.) injections of 0.1 ml allantoic fluid containing influenza A/Port Chalmers/1/73 (H3N2) ($10^7$ infectious units $ml^{-1}$) kindly provided by Solvay Pharmaceuticals (Weesp, The Netherlands). Two weeks later, splenocytes from the immunized mice were re-stimulated in vitro for 5 days with influenza-infected syngeneic spleen cells or with influenza-infected NB41A3 cells at an effector:stimulator ratio of 5:1. Spleen cells were collected and assayed for cytotoxic activity against influenza virus in a 4-h $^{51}$Cr-release assay.

### Cytotoxicity assays

One million influenza-infected and non-infected NB41A3 cells were labelled with 20μCi $Na_2{}^{51}CrO_4$ (Amersham, UK) for 1 h. These labelled target cells ($10^4$ in 0.05 ml medium per well) were then cocultured with serial dilutions of effector cells (0.1 ml per well) in round-bottomed microtiter wells. After 4 h incubation at 37°C, 0.1 ml supernatant was collected for γ-radiation counting. Medium with or without 0.5% Triton X-100 (Sigma) was used for the determination of maximal release or spontaneous release respectively. The percentage specific lysis was calculated as [(experimental release – spontaneous release)/(maximal release – spontaneous release)]× 100%. Where indicated, cytolytic activity was blocked by including saturating concentrations of anti-MHC I $D^d$ or anti-MHC I $K^k$ during labelling of target cells with $Na_2{}^{51}CrO_4$. Mean radioactivity was determined from triplicate cultures.

### RESULTS

### Expression of MHC class I molecules $D^d$ and $K^k$ on NB41A3 cells

FACScan analyses were performed to evaluate the expression of MHC class I molecules $D^d$ and $K^k$ on NB41A3. Under physiological conditions in the absence of IFN-γ, MHC class I $D^d$ and $K^k$

© 2000 Cancer Research Campaign

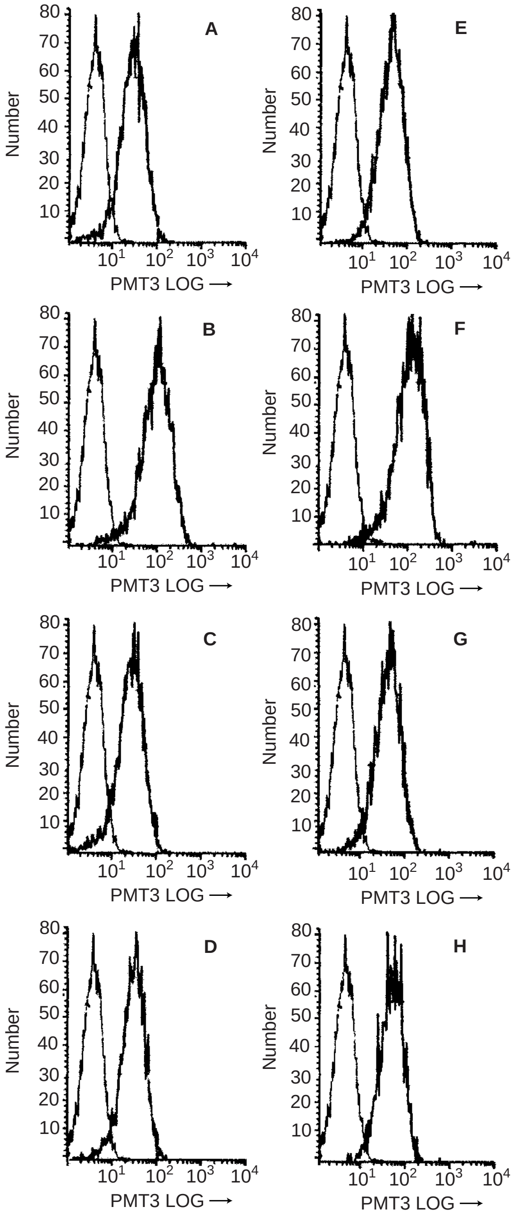

**Figure 1** Expression of MHC class I molecules on NB41A3 cells. NB41A3 cells were either untreated (**A** and **E**), treated with 750 U ml$^{-1}$ IFN-γ for 3 days (**B** and **F**), or infected with influenza virus for 2.5 h (**C** and **G**) or 6 h (**D** and **H**) and then incubated with monoclonal antibodies to MHC class I D$^d$ (**A**–**D**) or MHC class I K$^k$ (**E**–**H**). Cells were stained with a secondary FITC-conjugated rabbit anti-(mouse IgG) antibody and then analysed on the fluorescence-activated cell sorter. Fluorescence intensity was plotted vs. the cell number. Control cells (curves on the left in each panel) were stained with the secondary antibody only

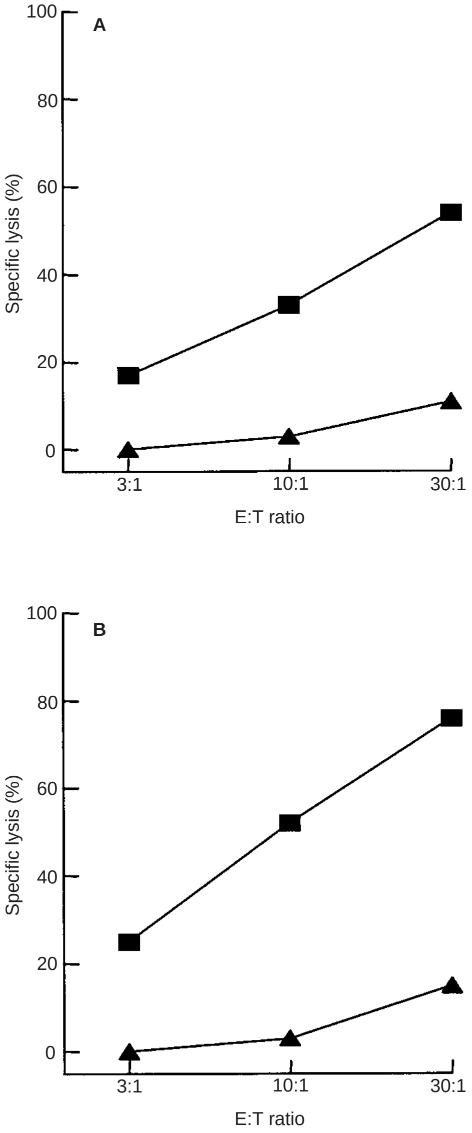

**Figure 2** Susceptibility of influenza-infected NB41A3 cells to influenza-specific CTLs. Influenza-specific CTLs were prepared by in vivo immunization of A/J mice with allantois fluid containing influenza virus Port Chalmers followed by in vitro restimulation with influenza-infected splenocytes as described in Material and Methods. Cytotoxic responses were measured at different effector-to-target ratios in a 4-h $^{51}$Cr-release assay. Susceptibility of non-stimulated NB41A3 cells (**A**) was compared with that of IFN-γ-stimulated NB41A3 cells (**B**). ▲, NB41A3 cells; ■, influenza-infected NB41A3 cells molecules were expressed to low and moderate extents respectively, as compared to their expression on P815 (H-2D$^d$) or L929 (H-2K$^k$) cells (Table 1). Treatment with IFN-γ (750 U ml$^{-1}$) for 3 days induced an up-regulation of the expression of both D$^d$ and K$^k$.

Influenza virus infection is known to induce IFN-α and IFN-β which in turn can induce up-regulation of MHC class I molecules (Halloran et al, 1989). We therefore evaluated the effect of acute influenza virus infection on expression of MHC class I molecules by staining NB41A3 cells 2.5 or 6 h post-infection. As shown in Figure 1, influenza virus infection of NB41A3 cells did not affect the expression of either MHC class I molecule on NB41A3 cells.

### MHC class I-restricted lysis of infected NB41A3 cells by influenza-specific CTLs

To investigate the antigen-presenting capacities, NB41A3 cells were infected with influenza virus A/Port Chalmers/1/73 (H3N2) and their susceptibility to killing by influenza-specific CTLs was determined. Influenza-specific CTLs were generated in A/J mice by i.p. immunization with $10^6$ infectious units of Port Chalmers virus. As demonstrated in Figure 2A, virus-infected NB41A3 cells were efficiently lysed by influenza-specific CTLs (58%

© 2000 Cancer Research Campaign

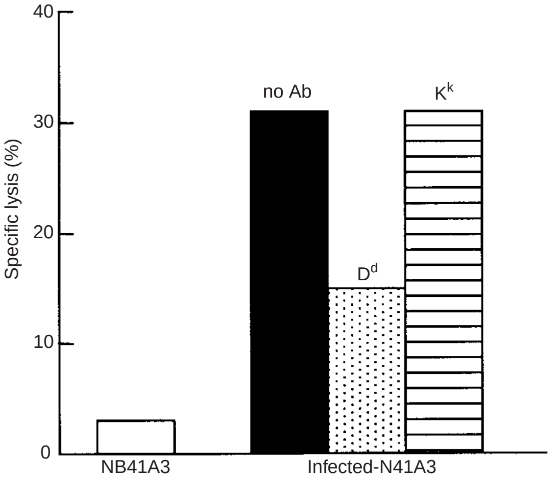

**Figure 3** Inhibition of CTL-mediated lysis of infected NB41A3 target cells by antibodies to MHC class I molecules. Influenza-specific CTLs were prepared as described before. Cytotoxic activity at a 30:1 effector-to-target ratio was determined in a 4-h $^{51}$Cr-release assay following an incubation of the target cells with saturating amounts of antibodies (60 min at 37°C). Antibodies used were anti-MHC I $D^d$ or anti-MHC I $K^k$

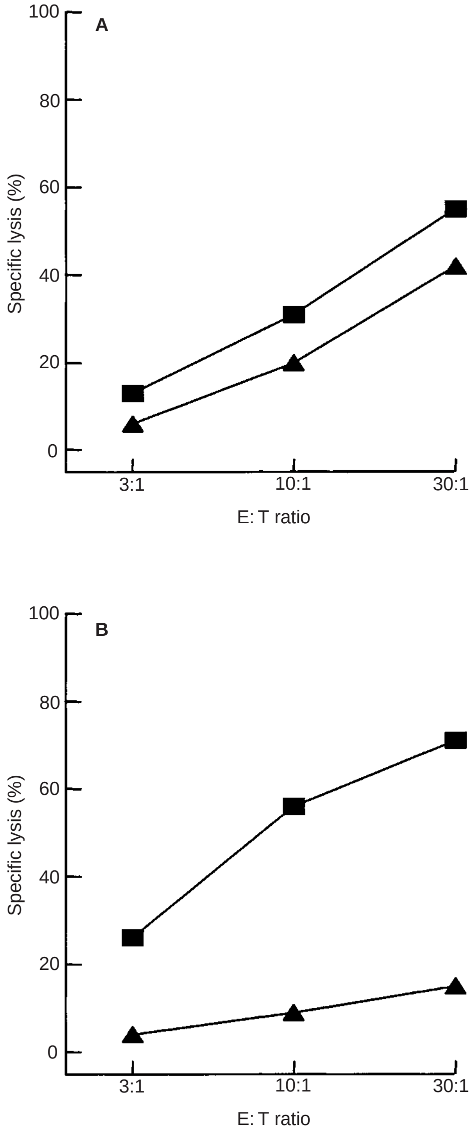

**Figure 4** Evaluation of the CTL-stimulating capacities of influenza-infected NB41A3 cells. Influenza-primed CTLs were restimulated in vitro with influenza-infected splenocytes (**A**) or influenza-infected NB41A3 cells (**B**). Cytotoxic responses were measured at different effector-to-target ratios in a 4-h $^{51}$Cr-release assay. (▲) NB41A3 cells; (■) influenza-infected NB41A3 cells specific lysis at E:T ratio 30:1). The sensitivity to lysis was increased to 78% when NB41A3 cells were stimulated with IFN-γ for 3 days prior to the $^{51}$Cr-release assay (Figure 2B).

To identify possible H-2 restriction elements, the ability of appropriate antibodies to inhibit lysis of influenza-infected NB41A3 cells by influenza-specific CTLs was determined. As demonstrated in Figure 3, lysis of infected NB41A3 cells was partially inhibited by antibodies against MHC I $D^d$, but not by antibodies against $K^k$ (48% and 0% inhibition respectively).

### In vitro stimulation of influenza-primed CTLs by infected NB41A3 cells

In order to test the ability of NB41A3 cells to stimulate spleen cells in vitro, bulk T cells of mice primed with Port Chalmers virus were incubated with virus-infected NB41A3 cells for 5 days. Cytotoxic activity of these T cells against infected targets was compared to that of T cells stimulated by influenza-infected spleen cells. As shown in Figure 4, infected NB41A3 cells could efficiently stimulate influenza-primed CTLs in vitro. Both splenocyte- and NB41A3-stimulated CTLs showed a similar level of specific lysis of influenza-infected target cells. Interestingly, lysis of non-infected targets being rather high for splenocyte-stimulated CTLs in this experiment (Figure 4A), was considerably lower when restimulation was performed with infected NB41A3 cells (Figure 4B).

### DISCUSSION

In the present study, we used influenza virus as a tool to determine the antigen presenting capacities of NB tumour cells. Clearly, influenza-infected NB41A3 cells can be recognized and lysed in vitro by influenza-specific CTLs only if the cells process and present viral antigens properly. Thus, the influenza assay measures three important steps in the MHC class I processing pathway, e.g. (i) processing of influenza-derived proteins to presentable peptides; (ii) transport of these peptides from the cell cytosol to the ER by TAP-1/TAP-2; (iii) presentation of the peptides in context of MHC class I molecules on the cell surface. Therefore, this functional assay evaluates not only the level of cell surface expression of MHC class I molecules, but also defects in the function of the proteasome subunits LMP2/LMP7 and the peptide-transporters TAP-1/TAP-2.

In addition, the influenza virus-based assay also measures the susceptibility of tumour cells for lysis by CTLs. T cells can cause the destruction of target cells by two mechanisms, one involving the death-signalling receptor CD95 (also called APO-1 or Fas) and the other involving the perforin-granzyme system. Both mechanisms eventually activate the caspase cascade leading to apoptosis (Froelich et al, 1996; Nagata, 1997). According to Strand and Galle (1998), an impaired anti-tumour immune response can be caused by reduced tumour-cell responsiveness to stimulation of CD95. In fact, many cancer cells are relatively resistant to CD95-mediated apoptosis because of loss of CD95 (no triggering of T-cell-induced apoptosis) or defects in the CD95 signalling pathway (no response to T-cell-induced apoptosis). Furthermore, many tumour cells overexpress apoptosis inhibitors such as Bcl-2 and Bcl-2 family members, which abrogate not only CD95-induced but also perforin-mediated apoptosis. The presented influenza virus-based assay can be used to evaluate the presence of such apoptosis-inhibiting mechanisms and can help to demonstrate susceptibility of tumour cells to CTL-mediated lysis in general.

To test the influenza virus-based method we used the murine neuroblastoma (NB) C1300, a well-established model for human disease (Ziegler et al, 1997). NB is a typical poorly immunogenic tumour and incapable to elicit a specific immune response by virtue of a low MHC class I expression and its deficiency in MHC class II, costimulatory and adhesion molecules (Lampson et al, 1983; Main et al, 1985; Zier et al, 1990; Katsanis et al, 1995). The low expression of MHC class I molecules was confirmed in this study. MHC class I down-regulation in NB cells is probably caused by overexpression of the N-*myc* oncogene which lowers the expression of the p50 subunit of NF-κB, a transcription factor binding to the enhancer-A element in the MHC class I gene promoter (Bernards et al, 1986; Lenardo et al, 1989; van't Veer et al, 1993).

In the context of the antigen-presentation assay, influenza-infected NB cells were efficiently recognized and lysed by influenza-specific CTLs despite the low expression of the relevant MHC class I molecules. Lysis was not due to MHC class I up-regulation by virus infection since infected NB cells showed no differences in expression of these molecules in comparison with non-infected NB cells. Although an up-regulated expression of MHC class I molecules by IFN-γ stimulation slightly increased the sensitivity of NB tumour cells to lysis, the limited number of MHC class I molecules expressed under physiological conditions appeared to be sufficient for efficient recognition by CTLs.

CTL-mediated killing of influenza-infected NB cells appeared to be restricted by H-2D$^d$ as shown by the fact that antibodies to H-2D$^d$ but not antibodies to H-2K$^k$ could reduce lysis. Interestingly, although viral nucleoproteins (NP) are often immunodominant targets for CTLs (Townsend et al, 1984; Yewdell et al, 1985), a conserved H-2K$^k$-binding epitope (AA50–57: SDYE-GRLI), derived from influenza NP (Parker and Gould, 1996) does obviously not play an important role in the killing of infected NB cells. Limited importance of an immunodominant NP-derived epitope was found earlier for peptide TYQRTALV (AA147–155) of influenza A/Puerto Rico/8/34 (H1N1). CTLs restimulated with influenza-infected cells, thus with a number of epitopes simultaneously, failed to recognize target cells which had been loaded with this NP peptide only (own observations). With respect to lysis of the NB cells, unknown H-2D$^d$-specific epitopes derived from NP, the matrix protein (M), or, perhaps, haemagglutinin (HA) were apparently immunodominant since lysis was restricted by H-2D$^d$. Unfortunately, influenza virus strain A/Port Chalmers/1/73 (H3N2) is poorly characterized as far as H-2-binding epitopes are concerned.

Besides being targets for influenza-specific CTLs, infected NB cells were also capable of stimulating bulk spleen cells in vitro. Stimulated spleen cells from influenza-primed mice, but not from PBS-injected control mice, could efficiently lyse influenza-infected target cells. It might be argued that stimulation was not mediated by the primarily infected NB41A3 cells but by secondarily infected spleen cells. Indeed, infected NB cells used as stimulators will produce virus. However, due to lack of a proper trypsin-like protease, cleavage of the viral haemagglutinin does not take place rendering the produced virus uninfectious (Klenk et al, 1975; Lazarowicz and Choppin, 1975). Therefore, stimulation of T cells by cells other than the initially infected NB cells can be excluded.

In summary, the present study demonstrates that the poorly immunogenic NB cells: (i) do possess a functional antigen-processing machinery, (ii) present antigen despite a low surface expression of the relevant MHC class I molecules, and (iii) are susceptible to lysis by activated CTLs. The efficient lysis of NB cells in this assay implies that NBs are suitable targets for immunotherapy if an effective way of immunization can be found to activate NB-specific CTLs. This paper presents a reliable and widely applicable functional assay using the influenza virus as a tool to determine the antigen processing and presenting capacities of tumour cells and to test their susceptibility to lysis by activated CTLs.

### ACKNOWLEDGEMENTS

We thank Mr G Mesander of the Cytometry Centre (University of Groningen, the Netherlands) for his assistance with the FACS analysis. This study was supported by the European Community Biotechnology Programme.

### REFERENCES

Bernards R, Dessain SK and Weinberg RA (1986) N-myc amplification causes down-modulation of MHC class I antigen expression in neuroblastoma. *Cell* **47**: 667–674

Christinck ER, Luscher MA, Barber BH and Williams DB (1991) Peptide binding to class I MHC on living cells and quantitation of complexes required for CTL lysis. *Nature* **352**: 67–70

Darrow TL, Slingluff CL Jr and Seigler HF (1989) The role of HLA class I antigens in recognition of melanoma cells by tumor-specific cytotoxic T lymphocytes. Evidence for shared tumour antigens. *J Immunol* **142**: 3329–3335

Demotz S, Grey HM and Sette A (1990) The minimal number of class II MHC-antigen complexes needed for T cell activation. *Science* **249**: 1028–1030

Ferrone S and Marincola FM (1995) Loss of HLA class I antigens by melanoma cells: molecular mechanisms, functional significance and clinical relevance. *Immunol Today* **16**: 487–494

Froelich CJ, Orth K, Turbov J, Seth P, Gottlieb R, Babior B, Shah GM, Bleackley RC, Dixit VM and Hanna W (1996) New paradigm for lymphocyte granule-mediated cytotoxicity. Target cells bind and internalize granzyme B, but an endosomolytic agent is necessary for cytosolic delivery and subsequent apoptosis. *J Biol Chem* **271**: 29073–29079

Garrido F, Cabrera T, Concha A, Glew S, Ruiz-Cabello F and Stern PL (1993) Natural history of HLA expression during tumour development. *Immunol Today* **14**: 491–499

Halloran PF, Urmson J, van der Meide PH and Autenried P (1989) Regulation of MHC expression in vivo. II. IFN-alpha/beta inducers and recombinant IFN-alpha modulate MHC antigen expression in mouse tissues. *J Immunol* **142**: 4241–4247

Harding CV and Unanue ER (1990) Quantitation of antigen-presenting cell MHCII/peptide complexes necessary for T-cell stimulation. *Nature* **346**: 574–576

Hicklin DJ, Marincola FM and Ferrone S (1999) HLA class I antigen downregulation in human cancers: T cell immunotherapy revives an old story. *Mol Med Today* **5**: 178–186

Katsanis E, Xu Z, Bausero MA, Dancisak BB, Gorden KB, Davis G, Gray GS, Orchard PJ and Blazar BR (1995) B7-1 expression decreases tumorigenicity and induces partial systemic immunity to murine neuroblastoma deficient in major histocompatibility complex and costimulatory molecules. *Cancer Gene Ther* **2**: 39–46

 © 2000 Cancer Research Campaign

Khanna R (1998) Tumour surveillance: missing peptides and MHC molecules. *Immunol Cell Biol* **76**: 20–26

Klenk HD, Rott R, Orlich M and Blodorn J (1975) Activation of influenza A viruses by trypsin treatment. *Virology* **68**: 426–439

Lampson LA, Fisher CA and Whelan JP (1983) Striking paucity of HLA-A, B, C and $\beta_2$-microaglobulin on human neuroblastoma cell lines. *J Immunol* **130**: 2471–2478

Lazarowitz SG and Choppin PW (1975) Enhancement of the infectivity of influenza A and B viruses by proteolytic cleavage of the hemagglutinin polypeptide. *Virology* **68**: 440–454

Lenardo M, Rustgi AK, Schievella AR and Bernards R (1989) Suppression of MHC class I gene by N-myc through enhancer inactivation. *EMBO J* **8**: 3351–3355

Main EK, Lampson LA, Hart MK, Kornbluth J and Wilson DB (1985) Human neuroblastoma cell lines are susceptible to lysis by natural killer cells but not by cytotoxic T lymphocytes. *J Immunol* **135**: 242–246

Marincola FM, Shamamian P, Simonis TB, Abati A, Hackett J, O'Dea T, Fetsch P, Yannelli J, Restifo NP, Mulé JJ and Rosenberg SA (1994*a*) Locus-specific analysis of human leukocyte antigen class I expression in melanoma cell lines. *J Immunother Emphasis Tumour Immunol* **16**: 13–23

Marincola FM, Shamamian P, Alexander RB, Gnarra JR, Turetskaya RL, Nedospasov SA, Simonis TB, Taubenberger JK, Yannelli J, Mixon A, Restifo NP, Herlyn M and Rosenberg SA (1994*b*) Loss of HLA haplotype and B locus down-regulation in melanoma cell lines. *J Immunol* **153**: 1225–1237

Nagata S (1997) Apoptosis by death factor. *Cell* **88**: 355–365

Parker CE and Gould KG (1996) Influenza A virus – a model for viral antigen presentation to cytotoxic T lymphocytes. *Semin Virol* **7**: 61–73

Restifo NP, Esquivel F, Kawakami Y, Yewdell JW, Mulé JJ, Rosenberg SA and Bennink JR (1993) Identification of human cancers deficient in antigen processing. *J Exp Med* **177**: 265–272

Rivoltini L, Barracchini KC, Viggiano V, Kawakami Y, Smith A, Mixon A, Restifo NP, Topalian SL, Simonis TB, Rosenberg SA and Marincola FM (1995) Quantitative correlation between HLA class I allele expression and recognition of melanoma cells by antigen-specific cytotoxic T lymphocytes. *Cancer Res* **55**: 3149–3157

Seliger B, Maeurer MJ and Ferrone S (1997) TAP off – tumors on. *Immunol Today* **18**: 292–299

Strand S and Galle PR (1998) Immune evasion by tumours: involvement of the CD95 (APO-1/Fas) system and its clinical implications. *Mol Med Today* **4**: 63–68

Sykulev Y, Joo M, Vturina I, Tsomides TJ and Eisen HN (1996) Evidence that a single peptide–MHC complex on a target cell can elicit a cytolytic T cell response. *Immunity* **4**: 565–571

Townsend ARM, McMichael AJ, Carter NP, Huddlesoton JA and Brownlee GG (1984) Cytotoxic T cell recognition of the influenza nucleoprotein and hemagglutinin expressed in transfected mouse L cells. *Cell* **39**: 13–25

Van't Veer LJ, Beijersbergen RL and Bernards R (1993) N-*myc* suppresses major histocompatibility complex class I gene expression through down-regulation of the p50 subunit of NF-κB. *EMBO J* **12**: 195–200

Yewdell JW, Bennink JR, Smith GL and Moss B (1985) Influenza A virus nucleoprotein is a major target antigen for cross reactive anti-influenza A virus cytotoxic T lymphocytes. *Proc Natl Acad Sci USA* **82**: 1785–1789

Ziegler MM, Ishizu H, Nagabuchi E, Takada N and Arya G (1997) A comparative review of the immunobiology of murine neuroblastoma and human neuroblastoma. *Cancer* **79**: 1757–1766

Zier KS, Pierson GR and Brown V (1990) Susceptibility of human neuroblastoma cell lines to cytotoxic T lymphocyte-mediated lysis. *J Neuroimmunol* **28**: 153–160

*© 2000 Cancer Research Campaign*

## References

[PDF_00592] Bernards R., Dessain S. K., Weinberg R. A. (1986). N-myc amplification causes down-modulation of MHC class I antigen expression in neuroblastoma.. Cell.

[PDF_00595] Christinck E. R., Luscher M. A., Barber B. H., Williams D. B. (1991). Peptide binding to class I MHC on living cells and quantitation of complexes required for CTL lysis.. Nature.

[PDF_00598] Darrow T. L., Slingluff C. L., Seigler H. F. (1989). The role of HLA class I antigens in recognition of melanoma cells by tumor-specific cytotoxic T lymphocytes. Evidence for shared tumor antigens.. J Immunol.

[PDF_00601] Demotz S., Grey H. M., Sette A. (1990). The minimal number of class II MHC-antigen complexes needed for T cell activation.. Science.

[PDF_00603] Ferrone S., Marincola F. M. (1995). Loss of HLA class I antigens by melanoma cells: molecular mechanisms, functional significance and clinical relevance.. Immunol Today.

[PDF_00606] Froelich C. J., Orth K., Turbov J., Seth P., Gottlieb R., Babior B., Shah G. M., Bleackley R. C., Dixit V. M., Hanna W. (1996). New paradigm for lymphocyte granule-mediated cytotoxicity. Target cells bind and internalize granzyme B, but an endosomolytic agent is necessary for cytosolic delivery and subsequent apoptosis.. J Biol Chem.

[PDF_00611] Garrido F., Cabrera T., Concha A., Glew S., Ruiz-Cabello F., Stern P. L. (1993). Natural history of HLA expression during tumour development.. Immunol Today.

[PDF_00614] Halloran P. F., Urmson J., Van der Meide P. H., Autenried P. (1989). Regulation of MHC expression in vivo. II. IFN-alpha/beta inducers and recombinant IFN-alpha modulate MHC antigen expression in mouse tissues.. J Immunol.

[PDF_00618] Harding C. V., Unanue E. R. (1990). Quantitation of antigen-presenting cell MHC class II/peptide complexes necessary for T-cell stimulation.. Nature.

[PDF_00621] Hicklin D. J., Marincola F. M., Ferrone S. (1999). HLA class I antigen downregulation in human cancers: T-cell immunotherapy revives an old story.. Mol Med Today.

[PDF_00624] Katsanis E., Xu Z., Bausero M. A., Dancisak B. B., Gorden K. B., Davis G., Gray G. S., Orchard P. J., Blazar B. R. (1995). B7-1 expression decreases tumorigenicity and induces partial systemic immunity to murine neuroblastoma deficient in major histocompatibility complex and costimulatory molecules.. Cancer Gene Ther.

[PDF_00629] Khanna R. (1998). Tumour surveillance: missing peptides and MHC molecules.. Immunol Cell Biol.

[PDF_00631] Klenk H. D., Rott R., Orlich M., Blödorn J. (1975). Activation of influenza A viruses by trypsin treatment.. Virology.

[PDF_00633] Lampson L. A., Fisher C. A., Whelan J. P. (1983). Striking paucity of HLA-A, B, C and beta 2-microglobulin on human neuroblastoma cell lines.. J Immunol.

[PDF_00637] Lazarowitz S. G., Choppin P. W. (1975). Enhancement of the infectivity of influenza A and B viruses by proteolytic cleavage of the hemagglutinin polypeptide.. Virology.

[PDF_00640] Lenardo M., Rustgi A. K., Schievella A. R., Bernards R. (1989). Suppression of MHC class I gene expression by N-myc through enhancer inactivation.. EMBO J.

[PDF_00642] Main E. K., Lampson L. A., Hart M. K., Kornbluth J., Wilson D. B. (1985). Human neuroblastoma cell lines are susceptible to lysis by natural killer cells but not by cytotoxic T lymphocytes.. J Immunol.

[PDF_00649] Marincola F. M., Shamamian P., Alexander R. B., Gnarra J. R., Turetskaya R. L., Nedospasov S. A., Simonis T. B., Taubenberger J. K., Yannelli J., Mixon A. (1994). Loss of HLA haplotype and B locus down-regulation in melanoma cell lines.. J Immunol.

[PDF_00645] Marincola F. M., Shamamian P., Simonis T. B., Abati A., Hackett J., O'Dea T., Fetsch P., Yannelli J., Restifo N. P., Mulé J. J. (1994). Locus-specific analysis of human leukocyte antigen class I expression in melanoma cell lines.. J Immunother Emphasis Tumor Immunol.

[PDF_00653] Nagata S. (1997). Apoptosis by death factor.. Cell.

[PDF_00656] Restifo N. P., Esquivel F., Kawakami Y., Yewdell J. W., Mulé J. J., Rosenberg S. A., Bennink J. R. (1993). Identification of human cancers deficient in antigen processing.. J Exp Med.

[PDF_00659] Rivoltini L., Barracchini K. C., Viggiano V., Kawakami Y., Smith A., Mixon A., Restifo N. P., Topalian S. L., Simonis T. B., Rosenberg S. A. (1995). Quantitative correlation between HLA class I allele expression and recognition of melanoma cells by antigen-specific cytotoxic T lymphocytes.. Cancer Res.

[PDF_00664] Seliger B., Maeurer M. J., Ferrone S. (1997). TAP off--tumors on.. Immunol Today.

[PDF_00666] Strand S., Galle P. R. (1998). Immune evasion by tumours: involvement of the CD95 (APO-1/Fas) system and its clinical implications.. Mol Med Today.

[PDF_00669] Sykulev Y., Joo M., Vturina I., Tsomides T. J., Eisen H. N. (1996). Evidence that a single peptide-MHC complex on a target cell can elicit a cytolytic T cell response.. Immunity.

[PDF_00672] Townsend A. R., McMichael A. J., Carter N. P., Huddleston J. A., Brownlee G. G. (1984). Cytotoxic T cell recognition of the influenza nucleoprotein and hemagglutinin expressed in transfected mouse L cells.. Cell.

[PDF_00678] Yewdell J. W., Bennink J. R., Smith G. L., Moss B. (1985). Influenza A virus nucleoprotein is a major target antigen for cross-reactive anti-influenza A virus cytotoxic T lymphocytes.. Proc Natl Acad Sci U S A.

[PDF_00681] Ziegler M. M., Ishizu H., Nagabuchi E., Takada N., Arya G. (1997). A comparative review of the immunobiology of murine neuroblastoma and human neuroblastoma.. Cancer.

[PDF_00684] Zier K. S., Pierson G. R., Brown V. (1990). Susceptibility of human neuroblastoma cell lines to cytotoxic T lymphocyte-mediated lysis.. J Neuroimmunol.

[PDF_00675] van 't Veer L. J., Beijersbergen R. L., Bernards R. (1993). N-myc suppresses major histocompatibility complex class I gene expression through down-regulation of the p50 subunit of NF-kappa B.. EMBO J.

